# Applying an Evolutionary Approach of Risk-Taking Behaviors in Adolescents

**DOI:** 10.3389/fpsyg.2021.694134

**Published:** 2022-01-10

**Authors:** Javier Salas-Rodríguez, Luis Gómez-Jacinto, Isabel Hombrados-Mendieta, Natalia del Pino-Brunet

**Affiliations:** Department of Social Psychology, Social Work, Social Anthropology and East Asian Studies, University of Málaga, Málaga, Spain

**Keywords:** evolutionary specific domain, risk-taking behavior, risk-return framework, adolescence, sex differences

## Abstract

Risk-taking behaviors in adolescents have traditionally been analyzed from a psychopathological approach, with an excessive emphasis on their potential costs. From evolutionary theory we propose that risk-taking behaviors can be means through which adolescents obtain potential benefits for survival and reproduction. The present study analyses sex differences in three contexts of risk (i.e., risk propensity, expected benefits and risk perception) in the evolutionary specific domains and the predictive value of these domains over risk-taking behaviors, separately in female and male adolescents. 749 adolescents (females = 370) valued their risk perception, expected benefits and risk propensity through the Evolutionary Domain-Specific Risk Scale, as well as their engagement in risk-taking behaviors through the Risky Behavior Questionnaire. Male adolescents showed lower risk perception in two evolutionary domains, expected higher benefits in two other domains and showed higher risk propensity in six domains. Female adolescents showed lower risk perception in two domains. Additionally, risk perception, expected benefits and risk propensity in the evolutionary domains predicted the engagement in risk-taking behaviors in male adolescents, whereas in female adolescents only expected benefits and risk propensity showed a predictive effect over risk-taking behaviors. These results suggest the potential role of evolutionary mechanisms on risk-taking behaviors in adolescents. Results have practical implications for interventions programs aimed at reducing risk-taking behaviors. In addition to considering sex differences, intervention programs should consider alternative behaviors through which adolescents can reach their evolutionary goals, and handle the risks related to those behaviors that cannot be replaced but have potential benefits for adolescents.

## Introduction

Adolescence and young adulthood are the developmental stages more related to risk-taking behaviors and the moment when several of these behaviors initiate (Steinberg, 2008; Willoughby et al., 2021). The study of risk-taking behaviors has generally focused on analyzing the negative impact of such behaviors on the individual’s health and social integration (e.g., Campbell et al., 2020). This approach derives from considering risk-taking behaviors as bad developmental outcomes generated by stressful environments (Ellis et al., 2012). As a consequence, research has put too much emphasis on the costs, ignoring the potential benefits of risk-taking behaviors in adolescents and leading to a psychopathological view of these behaviors (Machluf and Bjorklund, 2015).

### The Adaptive Value of Risk-Taking Behaviors

In contrast to the psychopathological view, evolutionary psychology considers the functional value of risk-taking behaviors in adolescents as means for obtaining potential gains (Ellis et al., 2012). From an evolutionary psychology approach, the major function of adolescence is to develop reproductive status, which can be reached through attaining social status, controlling resources, mating success, and other fitness-relevant outcomes (Ellis, 2012). For this purpose, although they look very different, both social high-risk behaviors (i.e., aggressive behaviors, substance use or unsafe sexual practices) and social low-risk behaviors (i.e., cooperation, reciprocation) may have the same functional value for attaining these immediate objectives (Ellis, 2012). Therefore, and despite their negative consequences, from an evolutionary perspective it is key to analyze both the potential costs and benefits of risk-taking behaviors in adolescents. Analyzing what adolescents gain from taking part in such behaviors could explain why these behaviors are worth or necessary. However, it should be noted that promoting a rationale for risk-taking behaviors does not mean that evolutionary psychologists justify these behaviors. From an adaptive perspective, we propose that adolescents who take part in risk-taking behaviors need to be viewed in a different manner in order to understand them. In fact, risk-taking behaviors such as bullying have been seen to increase access to resources, such as food, social status, and mates (Volk et al., 2012; Farrell and Dane, 2020).

Evolutionary psychology understands the human mind as a set of specific functional mechanisms designed to resolve recurrent problems related to survival and reproduction (Tooby and Cosmides, 2005). Each evolutionary specific domain is defined as a motivational domain comprised by a set of inputs, contents, objects, outcomes, or actions on which a series of assessment procedures were designed to act based on them (Tooby and Cosmides, 2005). Therefore, mechanisms involved in mate selection will be functionally different from those involved in the resolution of a group conflict or in deciding to avoid food poisoning. The evolutionary specific domains aimed at solving evolutionary content problems are shown in Table 1. Moreover, in the present study we focused on a risk-return framework, where risk-perception, expected benefits, and risk propensity are proposed as key cognitive variables in risk-taking behaviors engagement (Weber et al., 2002). Additionally, the risk-return framework has demonstrated that risk taking is domain specific (Blais and Weber, 2006; Hanoch et al., 2006; Figner and Weber, 2011), which is in line with the evolutionary-specific domain approach. In this sense, bringing together both an evolutionary approach on the human mind and the domain specific risk approach, Wilke et al. (2014) developed the Evolutionary Domains-Specific Risk Scale (ERS), which measures risk-taking behaviors in ten evolutionary content domains. From this point of view, risk-taking behaviors are conceptualized as variations in payoff distributions within specific domains of adaptation, in accordance with the risk-return framework.

**TABLE 1:** Evolutionary specific domains of life.

Competition (Puts et al., 2016; Hess and Hagen, 2019)	More intense in males, contest competition involves the use of force to exclude same-sex competitors in mating opportunities. This predisposition of males toward competition is observed as a higher tendency of men toward fighting and physical aggression, larger body size and strength compared to females (sexual dimorphism), and the design of weapons mainly for inter-male competition. Additionally, inter-male competition drives males to create alliances for between-group competition over territory. Competition can also take place between members of the same group (within-group competition) and with the same intensity in both sexes, both for social resources (e.g., mates) and material resources (e.g., food). Although it can take place in form of physical aggression, within-group competition occurs mainly through indirect aggression; for instance, through negative gossip and conceal positive information to damage the reputation of the opponent.
Cooperation (Henrich and Muthukrishna, 2021)	Natural selection has shaped individuals’ minds to help them assess their degree of interdependence with others and use such assessments to motivate higher affiliation, personal concern, and support. As a result, humans are an ultrasocial species that depends to a great extent on others for their own survival and reproduction. Through cooperation, behaviors such as food sharing, mutual aid, or communal defense are promoted. Evolutionary mechanisms that promote cooperation are kinship, direct reciprocity (i.e., you scratch my back, I’ll scratch yours) reputation (i.e., indirect reciprocity), punishment (i.e., penalizing defectors), and signaling (i.e., punishing non-cooperators).
Status (Anderson et al., 2015)	Throughout evolutionary history, being respected by the members of the community has provided survival and reproductive benefits. Status implies respect, admiration, and voluntary deference toward an individual perceived as possessing instrumental social value. This means that and individual or the community will confer status to that individual that is considered to have the necessary abilities to reach his/her own goals. Hierarchies of status are, therefore, formed when a group of individuals confers higher status to some of them, thus placing them in higher positions. As a result, individuals tend to prefer social environments where higher status is achievable, and they tend to react aggressively or violently when their social status is at threatened.
Mate choice (Buss and Schmitt, 2019)	Humans can show preference toward monogamous long-term mating (more frequent in females) or toward promiscuous short-term mating (more frequent in males). Choosing one or the other strategy will depend on fitness-related circumstances such as family, culture and ecology contexts, the stage of life or the ovulatory cycle. According to the parental investment theory (Trivers, 1972), males show lower minimum mandatory parental investment in offspring than females, so they tend to be less exigent in their mate choice. Conversely, females show higher minimum mandatory parental investment than males and are, therefore, more exigent when it comes to mate choice. This is due to the reproductive costs being higher if they make the wrong choice. Additionally, males tend to prefer physically attractive mates in long-term relationships, while females tend to prefer mates with the ability and interest of providing resources (mainly related to status and prestige). In short-term relationships, males mainly benefit from higher numbers of sexual partners, while females’ short-term strategies are not oriented toward quantity, but toward finding males with high quality genetics (e.g., masculinity and/or facial symmetry).
Foraging (Rozin and Todd, 2016)	This domain is associated with obtaining food and water and distinguishing what is toxic from what is nutritive. Foraging involves searching and capturing food, and its preparation for consumption and consumption. As omnivores, humans had to learn which food is potentially edible and which is potentially toxic. For this purpose, humans had to process sensorial cues such as color, texture, taste and odor, or food that others have eaten. In western societies, food choices are based mainly in palatability and healthfulness (which reflects caloric and nutritional value) and price and convenience (reflecting opportunity costs and handling time).
Parenting (Schaller, 2018)	During their first years of life, children need the care of parents to grow up and reach reproductive age. For this purpose, humans have a range of evolutionary mechanisms that regulate parenting behaviors. These mechanisms activate when having children, but also in non-parents, for example, by the perception superficial signs of childhood both in humans and non-human animals (small nose, big eyes). This domain is specifically related to tenderness, and it promotes rejection of risk.
Kinship (Hames, 2016)	This domain derives from kin selection (Hamilton, 1964), which implies that help between individuals will take place when the benefits perceived are higher than the costs for the individual providing it. But it also depends on the degree of relatedness between both individuals. As a result, humans possess specific mechanisms addressed to differentiate between close and distant kin and non-kin. This affects social interactions to a great extent (e.g., sibling altruism, incest avoidance). Consequently, individuals tend to support more their own kin when the benefit perceived, or the costs are higher for the provider than for the receiver.
Habitat selection (Silverman and Choi, 2016)	Throughout its evolutionary history, humans have had to travel systematically from one place to another to find food, water, and shelter, avoid predators, socialize, mate and parent. However, and despite such benefits, the search of a new environment implies potential costs such as energy use and danger (e.g., predators or rival groups). Curiosity is the adaptive trait that promotes the search for new information and, therefore, the exploration of new environments that can include characteristics of fitness-related opportunities.

### Risk-Taking Behaviors in Adolescents From a Risk-Return Framework

In line with the risk-return framework, it has been demonstrated that adolescents consider both the risks and benefits of taking part in risk-taking behaviors (Gibbons et al., 2009; Maslowsky et al., 2011). With respect to risk perception, it has been found that lower risk perception is positively related to binge drinking, sexual risk-taking, criminal behaviors, extreme sports and financial risks (Kershaw et al., 2003; Reniers et al., 2016; Martínez-Montilla et al., 2020; Altikriti et al., 2021), though higher risk perception has also been found to relate positively to risk-taking behaviors like speeding and drink-driving (Hatfield and Fernandes, 2009). Nevertheless, expected benefits in risk-taking behaviors is more strongly associated with the engagement in these behaviors than risk perception in adolescents (Cauffman et al., 2010; Mantzouranis and Zimmermann, 2010). In fact, expected benefits predict engagement in risk-taking behaviors like drinking and smoking, sexual risk-taking, financial risk-taking, illicit substance use, and criminal activities (e.g., shoplifting, forgery and buying illegal drugs) (Parsons et al., 2000; Goldberg et al., 2002; Dhami and Mandel, 2012; Reniers et al., 2017; Carlson and Duckworth, 2019; Andrews et al., 2020; Hammond et al., 2020). Finally, risk-propensity has also been associated with higher engagement in risk-taking behaviors like substance use, unsafe sexual behavior, reckless driving, or delinquency in adolescents (Lejuez et al., 2003, 2007; Aklin et al., 2005; MacPherson et al., 2010). In essence, previous literature on risk-taking behaviors suggests the potential adaptive value of such behaviors, in line with the evolutionary approach on risk-taking behaviors (Ellis et al., 2012). More specifically, and despite perceiving potential costs, the influence of the expected benefits from taking part in such behaviors is higher for individuals, thus leading them to behave in such risky ways.

### Sex Differences in Risk-Taking Behaviors

In general, males take part in risk-taking behaviors to a greater degree than females (e.g., Byrnes et al., 1999; Archer, 2004, 2019, Luoto and Varella, 2021). These sex differences in risk-taking behaviors have been observed in hunter-gatherer societies (Apicella et al., 2017), which have represented the subsistence system during great part of our existence as species. Evolutionary psychology explains these sex differences in risk-taking behaviors as a result of the higher benefits males extract from these behaviors, compared to females (Trivers, 1972; Archer, 2019). Specifically, given the higher variation in male reproductive success compared to females, male reproductive competition (i.e., inter-male competition) tends to be more intense than female reproductive competition, which has led to a riskier male selection process (Archer, 2019). Based on this premise, Wilson and Daly (1985) coined the term *young male syndrome*, which is defined as a tendency toward higher engagement in risky and violent behaviors in the sex with the most intense reproductive competition in order to enhance social status. More recently, Fessler et al. (2014) proposed the *crazy bastard hypothesis*, on the basis of the *young male syndrome*, in reference to males’ higher involvement in voluntary risks as a means of signaling formidability. Along with the higher benefits males extract from taking part in risk-taking behaviors, females’ higher risk-aversion should be noted, given their fundamental role in offspring rearing (Campbell et al., 2021). These ideas were tested in an experimental study, which demonstrated that males expressed higher risk-taking behaviors in inter-male competition conditions, whereas females avoided to take risks in conditions of parental investment in young children (Fischer and Hills, 2012). Finally, from the costly signaling theory, risk-taking is proposed as an honest signaling mechanism through which males show their ability to take risks without suffering negative consequences (Bird et al., 2001; Prokop and Pazda, 2020). A recent study found that higher indices of embodied capital (i.e., attractiveness, intelligence, and dexterity) were positively related to several instantiations of risk-taking propensity (Refaie and Mishra, 2020). Nevertheless, females can also engage in risk-taking behaviors with the purpose of competing for mates with other females (Arnocky and Vaillancourt, 2017).

Sex differences in risk-taking behaviors have also been observed in adolescents. In particular, female adolescents tend to perceive risks related to risky driving, alcohol and substance use, or antisocial behavior more than male adolescents (Essau, 2004; Rundmo and Iversen, 2004; Grevenstein et al., 2015). By contrast, male adolescents tend to perceive the benefits of risk-taking behaviors like unsafe sexual practices, risky driving, or alcohol use more than female adolescents (Kershaw et al., 2003; Widdice et al., 2006; Rhodes and Pivik, 2011). Furthermore, several meta-analyses show that male adolescents are more risk-prone than female adolescents in several risk-taking behaviors (Byrnes et al., 1999; Archer, 2004). In short, these findings in adolescents are in line with the evolutionary suggestion that male adolescents gain higher advantages by taking part in risk taking-behaviors compared to female adolescents (Ellis et al., 2012; Archer, 2019).

### Present Study

Previous research shows sex differences in risk perception, expected benefits and risk propensity in adolescents and their predictive value over risk-taking behaviors. Nevertheless, and despite the adaptive value of risk-taking behaviors, no research has been carried out to analyze differences between male and female adolescents in the perception of risks and benefits, and the propensity to take risks in the evolutionary domains of risk. Therefore, one of the main objectives in present study was to analyze sex differences in risk perception, expected benefits and risk propensity in the evolutionary domains of risk in adolescents. Since intrasexual competition tends to be more intense in males, with females being more risk-averse (Trivers, 1972; Archer, 2019), we propose that male adolescents will tend to show higher expected benefits and risk propensity in the evolutionary domains compared to females; by contrast, females will show greater risk perception.

Furthermore, it was recently found that propensity to take risks in evolutionary domains relates to risk-taking behaviors in young adults (Salas-Rodríguez et al., 2021). Therefore, another objective in the present study was to analyze the predictive value of risk perception, expected benefits and risk propensity in the evolutionary domains over risk-taking behaviors, in male and female adolescents separately. Results could be key for establishing effective intervention programs designed to reduce dangerous behaviors such as aggression, substance use or unsafe sexual practices in adolescents according to each sex.

## Method

### Participants

The study was carried out in six education centers in the region of Málaga, Spain. 749 (females = 370) adolescent students from secondary education, college and vocational training took part in the study. Participants’ ages ranged between 13 and 18 years (*M* = 16.98; SD = 1.04). Prior to carrying out the study, parents and/or legal tutors of participants were informed about the objectives and methods of it. Participants also gave their verbal consent before answering the questionnaires. The present study was approved by the Ethical Committee on Experimentation from the University of Málaga (CEUMA) (Registry number: 45-2018-H).

### Instruments

#### Evolutionary Domain-Specific Risk Scale

The Spanish version of the Evolutionary Domain-Specific Risk Scale (ERS) was applied. The scale is comprised of 28 items which measure contemporary risk-taking behaviors that are analogous qualitatively of recurring problems in ancestral times which were relevant for survival and reproduction (Wilke et al., 2014). The ERS consists of ten domains: between-group competition (e.g., Adamantly defending the honor of your local team against a fan from a different sporting team, even if it may cause a fight), within-group competition (e.g., Trying to take a leadership role in any peer group you join), status/power (e.g., Blackmailing your opponent to win an election), environmental exploration (e.g., Swimming far out from shore to reach a diving platform), food selection (e.g., Eating only at restaurants that do not sell fast food), food acquisition (e.g., Eating food that has been dropped), parent-offspring conflict (e.g., Talking your parents into giving you weekly allowance money), kinship (e.g., Donating an organ to your sibling), mate attraction (e.g., Casually dating more than one person at a time), and mate retention (e.g., Spending the night with an attractive person while vacationing without your significant other). In the Spanish adapted version of the ERS, each domain comprises three items, except for the domain of food selection, which only comprises one item because it was considered that the content of the other two items in this domain was not assessable for adolescents. Therefore, items “Planting your own garden to grow your own fruits and vegetables” and “Significantly increasing your weekly food bill to buy healthy organic food” were removed from the final scale.

Moreover, the ERS can be handed for three different contexts: expected benefits, which measures the perceived benefits of engaging in each situation and with answering options ranging from (1) Not beneficial at all to (5) Very beneficial; risk perception, which measures the perceived risks of engaging in each situation and with answering options ranging from (1) Not risky at all to (5) Very risky; and risk propensity, which measures the likelihood of engaging in each situation, with answering options ranging from (1) Highly unlikely to (5) Very likely. Due to time limits imposed by school regarding the time consumed by handing the questionnaires, each participant only answered one of the three contexts of risk of the ERS. As a result, 230 participants (females = 119) answered the expected benefits context; 271 participants (females = 131) answered the risk perception context; and 248 participants (females = 120) answered the risk propensity context. Table 2 shows Cronbach values of the ERS.

**TABLE 2:** Cronbach values for each of the evolutionary domains in the three contexts of the ERS.

Domains	Risk perception	Expected benefits	Risk propensity
Between-group competition:	0.49	0.42	0.56
Within-group competition	0.47	0.52	0.59
Status/power:	0.70	0.57	0.58
Environmental exploration:	0.49	0.28	0.46
Food acquisition:	0.64	0.50	0.51
Food selection:	–	–	–
Parent/offspring conflict:	0.48	0.58	0.58
Kinship:	0.68	0.51	0.44
Mate attraction:	0.45	0.56	0.42
Mate retention:	0.24	0.31	0.40

*The domain of food selection comprised one item in the Spanish version of the ERS.*

#### Risky Behavior Questionnaire

The Spanish version of the Risky Behavior Questionnaire (RBQ) was applied in order to analyze the level of engagement of adolescents in risk-taking behaviors during the six previous months (Auerbach and Gardiner, 2012). The RBQ comprises 20 items which measure engagement in several types of risk-taking behaviors such as unsafe sexual practices, aggressive and/or violent behaviors, rule breaking, dangerous, destructive and illegal behaviors, self-injurious behaviors, and substance use. Although the RBQ comprises multiple dimensions, it has also been used as a single dimension of general engagement in risk-taking behaviors (e.g., Auerbach and Gardiner, 2012; Salas-Rodríguez et al., 2021). In the Spanish version of the RBQ, the answering format includes four answering options: (0) Never (not once); (1) Almost never (once a month); (2) Sometimes (2–3 times per month); and (3) Usually (3 or more times per week). In the present study, Cronbach value for the questionnaire was 0.83, similar to a previous study by Auerbach and Gardiner (2012), which showed α = 0.85.

#### Sociodemographic Questionnaire

Along with questions related to age and sex, participants answered a series of sociodemographic questions related to nationality (90.7% Spanish, 9.1% foreigners), relationship status (31.2% in a couple, 67.6% single), and current academic course (40.4% secondary education, 52.8% college, 6.3% vocational training).

### Procedure

Prior to the study, a pilot test was carried out in adolescents to verify the correct readability of items included in the RBQ and the ERS. Likewise, teachers from the different classrooms involved in the study were also asked about the level of readability of the items in both questionnaires. Upon completion of this initial stage, researchers went to the education centers to hand the questionnaires to students over 1 month. Participants answered self-report questionnaires in the classroom where they were taking lessons at that moment. Completing questionnaires lasted 25 min on average, with one of the researchers being present to resolve any doubts from participants. In all cases, participants answered the RBQ in the first place. Secondly, each participant was assigned randomly one of the three contexts of risk of the ERS (i.e., risk perception, expected benefits, or risk propensity) and finally, sociodemographic questions were included at the end of the instrument.

## Results

A multivariate analysis of variance (MANOVA) was carried out to check sex differences in risk perception, expected benefits and risk propensity in the ten evolutionary domains of risk and the engagement in risk-taking behaviors. Although most studies that analyze group differences in multiple dependent variables use multiple ANOVAs or multiple independent sample *t*-tests, both procedures increase the likelihood of falling into a Type I error, contrary to MANOVA (Warne, 2014). The Risk contexts of the ERS (three levels: risk perception, expected benefits, and risk propensity) and sex (two levels: males and females) were introduced as independent variables, and the 10 evolutionary domains of risk (ERS), along with risk-taking behaviors (RBQ), as dependent variables. Table 3 shows descriptive statistics for each of the ten evolutionary domains of risk as well as for risk-taking behaviors based on the risk context of the ERS (i.e., risk perception, expected benefits, and risk propensity) and sex. The MANOVA revealed a significant interaction effect between risk context and sex (Pillai’s Trace = 0.12, *F* = 4.085, *p* < 0.001, η^2^ = 0.060). Bonferroni *post hoc* tests showed sex differences in risk perception in four of the ten evolutionary domains. More specifically, female adolescents expressed higher risk perception in environmental exploration and kinship compared to male adolescents, whereas males perceived higher risks in parent-offspring conflict and mate retention. Regarding the context of expected benefits, sex differences were found in two evolutionary domains, with male adolescents perceiving more benefits in environmental exploration and mate attraction, compared to female adolescents. By contrast, female adolescents did not expect more benefits than male adolescents in any evolutionary domain. For risk propensity, six of the ten evolutionary domains showed differences between sexes. Male adolescents showed higher propensity to take risks in between-group competition, within-group competition, status/power, environmental exploration, food acquisition and mate attraction compared to female adolescents. Again, female adolescents did not show more propensity to take risks in any domain, compared to male adolescents. Finally, no sex differences were found in risk-taking behaviors measured with the RBQ in any of the risk contexts of the ERS (Table 3).

**TABLE 3:** Statistic descriptives and sex differences between risk contexts in the ten evolutionary domains and risk-taking behaviors.

	Risk perception				Expected benefits				Risk propensity			
	Males (*n* = 140)	Females (*n* = 131)	SE	95%CI	Males (*n* = 111)	Females (*n* = 119)	SE	95%CI	Males (*n* = 128)	Females (*n* = 120)	SE	95% CI
ERS												
Between-group competition	3.21 (0.96)	3.35 (0.91)	0.11	[−0.37, 0.07]	2.17 (0.90)	1.94 (0.73)	0.12	[0.00, 0.47]	2.69 (1.08)***	2.23 (0.86)***	0.12	[0.23, 0.69]
Within-group competition	2.76 (0.88)	2.71 (0.86)	0.10	[−0.15, 0.25]	2.35 (0.84)	2.13 (0.79)	0.11	[0.00, 0.43]	2.63 (0.81)**	2.33 (0.89)**	0.11	[0.09, 0.51]
Status/power	3.42 (1.02)	3.36 (1.11)	0.10	[−0.14, 0.26]	1.64 (0.80)	1.46 (0.58)	0.11	[−0.03, 0.40]	1.79 (0.79)***	1.39 (0.51)***	0.11	[0.19, 0.61]
Environmental exploration	2.94 (1.01)**	3.31 (0.86)**	0.11	[−0.59, −0.15]	2.72 (0.92)**	2.35 (0.74)**	0.12	[0.13, 0.61]	3.18 (1.00)***	2.72 (0.95)***	0.12	[0.23, 0.69]
Food selection	2.02 (1.32)	1.76 (1.10)	0.15	[−0.04, 0.55]	2.70 (1.21)	2.96 (1.27)	0.16	[−0.57, 0.06]	2.09 (1.22)	2.20 (1.25)	0.16	[−0.42, 0.19]
Food acquisition	3.39 (1.04)	3.33 (1.13)	0.11	[−0.15, 0.27]	1.60 (0.70)	1.40 (0.46)	0.12	[−0.02, 0.44]	2.16 (0.89)*	1.94 (0.82)*	0.11	[0.01, 0.44]
Parent-offspring	2.69 (0.90)*	2.40 (0.93)*	0.12	[0.06, 0.52]	2.63 (0.91)	2.71 (0.98)	0.13	[−0.33, 0.16]	2.67 (1.03)	2.64 (0.97)	0.12	[−0.20, 0.27]
Kinship	2.50 (1.17)***	2.89 (1.18)***	0.11	[−0.59, −0.17]	4.25 (0.67)	4.25 (0.69)	0.12	[−0.22, 0.23]	4.30 (0.69)	4.48 (0.54)	0.11	[−0.40, 0.04]
Mate attraction	3.08 (0.99)	3.27 (1.01)	0.11	[−0.40, 0.03]	2.39 (1.09)***	1.94 (0.72)***	0.12	[0.22, 0.69]	2.25 (0.89)**	1.89 (0.63)**	0.11	[0.14, 0.59]
Mate retention	3.18 (0.86)**	2.86 (0.85)**	0.09	[0.14, 0.51]	1.93 (0.79)	1.76 (0.62)	0.10	[−0.04, 0.36]	1.99 (0.81)	1.82 (0.67)	0.10	[−0.02, 0.37]
**RBQ**												
Risk-taking behaviors	0.51 (0.40)	0.49 (0.37)	0.04	[−0.07, 0.11]	0.50 (0.41)	0.53 (0.35)	0.05	[−0.13, 0.06]	0.50 (0.35)	0.45 (0.31)	0.05	[−0.05, 0.14]

*ERS responses were given on a five-point scale; RBQ responses were given on a four-point scale.*

*p ≤ 0.05; **p ≤ 0.01; ***p ≤ 0.001.

Correlation analyses were carried out to check associations between evolutionary domains of risk and risk-taking behaviors, separately for each risk context of the ERS and sex. Results can be seen on Table 4 (risk perception context), Table 5 (expected benefits context), and Table 6 (risk propensity context). Finally, multiple regression analysis with an enter method were carried out to analyze the predictive value of risk perception, expected benefits and risk propensity in the evolutionary domains over risk-taking behaviors, separately. These multiple regression analyses were also performed separately in female and male adolescents. It is necessary to point out that, although a predictive language is employed, correlational results must not be interpreted as causal effects. Regarding the context of risk perception, the multiple regression model was not statistically significant in females (Table 7). This means that the perception of risks in the evolutionary domains did not predict females’ engagement in risk-taking behaviors. On the other hand, the multiple regression model for the risk perception context was statistically significant in males, although it explained the lowest variance compared to the expected benefits and risk propensity models (Table 7). More specifically, perceiving higher risks in within-group competition and parent-offspring conflict predicted higher engagement in risk-taking behaviors in males, while perceiving higher risks in status/power showed the opposite effect. Regarding the context of expected benefits, regression models were statistically significant and showed the highest explained variance over risk-taking behaviors, both in females and males (Table 8). For females only expecting benefits in mate attraction predicted greater engagement in risk-taking behaviors. By contrast, in males, expected benefits in food acquisition and mate attraction predicted higher engagement in risk-taking behaviors. On the contrary, expected benefits in food selection predicted lower engagement in risk-taking behaviors in male adolescents. Finally, in the context of risk propensity, the multiple regression analysis model for females was statistically significant (Table 9): higher propensity to take risks in food acquisition and mate attraction predicted higher engagement in risk-taking behaviors in females. In the case of males, the multiple regression analysis model was also statistically significant (Table 9): higher risk propensity in between-group competition, status/power and mate attraction predicted higher engagement in risk-taking behaviors.

**TABLE 4:** Zero-order correlations between risk perception in the evolutionary-specific domains and risk-taking behaviors by sex.

	1	2	3	4	5	6	7	8	9	10	11
Between-group competition	−	0.54***	0.53***	0.28**	0.05	0.44***	0.31***	–0.10	0.33***	0.24**	–0.15
Within-group competition	0.45***	−	0.56***	0.22*	0.12	0.31***	0.33***	−0.28**	0.47***	0.41***	–0.03
Status/power	0.45***	0.44***	−	0.29**	0.07	0.49***	0.23**	−0.47***	0.54***	0.38***	–0.03
Environmental exploration	0.45***	0.28**	0.25**	−	0.13	0.35***	0.04	–0.06	0.24**	0.29**	–0.07
Food selection	–0.12	0.12	–0.00	–0.02	−	0.05	0.23**	0.07	–0.05	0.10	0.05
Food acquisition	0.42***	0.26**	0.48***	0.33***	0.03	−	0.10	−0.37***	0.46***	0.44***	–0.11
Parent-offspring	0.31***	0.29**	0.20*	0.25**	0.32***	0.26**	−	–0.11	0.09	0.08	0.05
Kinship	0.23**	0.10	−0.18*	0.24**	–0.04	–0.11	–0.08	−	−0.40***	–0.17	0.02
Mate attraction	0.41***	0.30***	0.46***	0.30***	0.14	0.31***	0.26**	–0.02	−	0.34***	–0.14
Mate retention	0.34***	0.29**	0.39***	0.19*	0.12	0.27**	0.22**	–0.04	0.49***	−	0.01
Risk-taking behaviors	−0.22**	–0.02	−0.38***	–0.15	0.01	−0.28**	0.05	0.12	−0.24**	–0.11	−

Above the diagonal: females (n = 131). Below the diagonal: males (n = 140). ***p < 0.001. **p < 0.01. *p < 0.05.

**TABLE 5:** Zero-order correlations between expected benefits in the evolutionary-specific domains and risk-taking behaviors by sex.

	1	2	3	4	5	6	7	8	9	10	11
Between-group competition	−	0.26**	0.37***	0.28**	–0.03	0.30**	0.23*	–0.02	0.16	0.34***	0.15
Within-group competition	0.30**	−	0.50***	0.23*	0.07	0.23*	0.32***	–0.01	0.18	0.26**	0.16
Status/power	0.46***	0.43***	−	0.25**	0.03	0.27**	0.23*	–0.11	0.12	0.23*	0.21*
Environmental exploration	0.44***	0.20*	0.25**	−	0.11	0.33***	0.21*	–0.01	0.08	0.22*	0.16
Food selection	–0.09	–0.01	–0.05	–0.08	−	–0.15	0.02	0.02	0.08	–0.07	–0.10
Food acquisition	0.36***	0.26**	0.35***	0.41***	−0.24*	−	0.22*	–0.17	0.04	0.27**	0.25**
Parent-offspring	0.26**	0.47***	0.45**	0.12	0.08	0.21*	−	–0.15	0.12	0.17	0.20*
Kinship	−0.29**	−0.34***	−0.35***	–0.08	0.12	–0.12	–0.10	−	–0.09	−0.22*	–0.16
Mate attraction	0.32**	0.49***	0.55***	0.11	0.02	0.25**	0.45***	−0.31**	−	0.14	0.38***
Mate retention	0.39***	0.38***	0.50***	0.22*	0.03	0.22*	0.27**	−0.23*	0.49***	−	0.16
Risk-taking behaviors	0.31**	0.25**	0.42***	0.22*	−0.24**	0.43***	0.19*	−0.33***	0.48**	0.35***	−

Above the diagonal: females (n = 119). Below the diagonal: males (n = 111). ***p < 0.001. **p < 0.01. *p < 0.05.

**TABLE 6:** Zero-order correlations between risk propensity in the evolutionary-specific domains and risk-taking behaviors by sex.

	1	2	3	4	5	6	7	8	9	10	11
Between-group competition	−	0.19*	0.17	0.19*	–0.04	0.19*	0.29**	–0.09	0.18	0.19*	0.01
Within-group competition	0.32***	−	0.41***	–0.07	–0.05	0.31**	0.40***	–0.16	0.35***	0.04	0.06
Status/power	0.44***	0.53***	−	0.19*	0.16	0.19*	0.26**	–0.17	0.31**	0.20*	0.22*
Environmental exploration	0.40***	0.30**	0.29**	−	0.08	0.28**	0.09	0.06	0.19*	0.29**	0.24**
Food selection	–0.03	0.15	0.20*	0.00	−	–0.11	–0.04	–0.10	–0.05	0.07	0.06
Food acquisition	0.19*	0.26**	0.23**	0.39***	0.10	−	0.33***	–0.02	0.05	0.07	0.24**
Parent-offspring	0.44**	0.39***	0.47***	0.16	0.17	0.34***	−	–0.15	0.19*	0.12	0.13
Kinship	0.19*	0.00	0.05	0.07	–0.05	–0.07	–0.04	−	−0.24**	–0.18	0.06
Mate attraction	0.25**	0.31***	0.38***	0.16	0.32***	0.14	0.29**	0.11	−	0.30**	0.30**
Mate retention	0.21*	0.14	0.29**	0.19*	0.25**	–0.03	0.19*	0.01	0.47***	−	0.18
Risk-taking behaviors	0.38***	0.16	0.40***	0.24**	0.16	0.14	0.19*	0.11	0.42***	0.27**	−

Above the diagonal: females (n = 120). Below the diagonal: males (n = 128). ***p < 0.001. **p < 0.01. *p < 0.05.

**TABLE 7:** Multiple regression analysis for risk perception-context predicting risk-taking behaviors.

	Males (*n* = 140)						Females (*n* = 131)					
Evolutionary Domain	*B*	*SE B*	95% CI	β	*t*	*p*	*B*	*SE B*	95% CI	β	*t*	*p*
Between-group competition	–0.07	0.05	[−0.16, 0.02]	–0.16	–1.43	0.15	–0.08	0.05	[−0.18, 0.02]	–0.20	–1.59	0.11
Within-group competition	0.09	0.04	[0.00, 0.17]	0.19	2.03	0.04	0.03	0.05	[−0.08, 0.13]	0.06	0.50	0.62
Status/power	–0.13	0.04	[−0.21, -0.04]	–0.32	–3.01	0.00	0.04	0.04	[−0.05, 0.13]	0.12	0.89	0.37
Environmental exploration	–0.03	0.04	[−0.10, 0.04]	–0.07	–0.79	0.43	–0.01	0.04	[−0.10, 0.07]	–0.03	–0.33	0.74
Food selection	–0.02	0.03	[−0.08, 0.03]	–0.08	–0.92	0.36	0.01	0.03	[−0.06, 0.07]	0.02	0.18	0.86
Food acquisition	–0.04	0.04	[−0.11, 0.03]	–0.11	–1.14	0.26	–0.02	0.04	[−0.09, 0.06]	–0.05	–0.38	0.71
Parent-offspring conflict	0.09	0.04	[0.01, 0.17]	0.20	2.17	0.03	0.03	0.04	[−0.05, 0.11]	0.07	0.72	0.47
Kinship	0.03	0.03	[−0.03, 0.09]	0.10	1.09	0.28	0.00	0.03	[−0.07, 0.07]	0.01	0.08	0.94
Mate attraction	–0.04	0.04	[−0.12, 0.04]	–0.09	–0.95	0.34	–0.06	0.04	[−0.14, 0.02]	–0.16	–1.43	0.16
Mate retention	0.03	0.04	[−0.05, 0.11]	0.06	0.70	0.49	0.03	0.05	[−0.06, 0.12]	0.07	0.61	0.54

*Males R^2^ = 0.23 (p = 0.00). Females R^2^ = 0.06 (p = 0.68).*

**TABLE 8:** Multiple regression analysis for expected benefit-context predicting risk-taking behaviors.

	Males (*n* = 111)						Females (*n* = 119)					
Evolutionary Domain	*B*	*SE B*	95% CI	β	*t*	*p*	*B*	*SE B*	95% CI	β	*t*	*p*
Between-group competition	0.00	0.04	[−0.09, 0.09]	0.00	–0.03	0.97	–0.01	0.05	[−0.11, 0.08]	–0.03	–0.29	0.78
Within-group competition	–0.05	0.05	[−0.14, 0.05]	–0.10	–1.04	0.30	–0.01	0.05	[−0.10, 0.08]	–0.03	–0.26	0.79
Status/power	0.05	0.06	[−0.06, 0.16]	0.09	0.88	0.38	0.07	0.06	[−0.06, 0.19]	0.11	1.05	0.29
Environmental exploration	0.01	0.04	[−0.07, 0.09]	0.03	0.32	0.75	0.03	0.04	[−0.06, 0.12]	0.06	0.66	0.51
Food selection	–0.06	0.03	[−0.11, 0.00]	–0.17	–2.05	0.04	–0.03	0.02	[−0.08, 0.01]	–0.12	–1.37	0.17
Food acquisition	0.15	0.05	[0.05, 0.26]	0.26	2.83	0.01	0.12	0.07	[−0.03, 0.26]	0.15	1.57	0.12
Parent-offspring conflict	–0.02	0.04	[−0.10, 0.07]	–0.04	–0.45	0.65	0.03	0.03	[−0.03, 0.10]	0.10	1.06	0.29
Kinship	–0.09	0.05	[−0.20, 0.01]	–0.15	–1.77	0.08	–0.04	0.05	[−0.13, 0.05]	–0.07	–0.84	0.40
Mate attraction	0.13	0.04	[0.05, 0.21]	0.35	3.32	0.00	0.17	0.04	[0.09, 0.26]	0.36	4.12	0.00
Mate retention	0.04	0.05	[−0.05, 0.14]	0.09	0.91	0.36	0.00	0.05	[−0.10, 0.11]	0.00	0.02	0.98

*Males R^2^ = 0.41 (p = 0.00). Females R^2^ = 0.24 (p = 0.00).*

**TABLE 9:** Multiple regression analysis for risk propensity-context predicting risk-taking behaviors.

	Males (*n* = 128)						Females (*n* = 120)					
Evolutionary Domain	*B*	*SE B*	95% CI	β	*t*	*p*	*B*	*SE B*	95% CI	β	*t*	*p*
Between-group competition	0.08	0.03	[0.02, 0.14]	0.24	2.48	0.01	–0.04	0.03	[−0.11, 0.03]	–0.11	–1.18	0.24
Within-group competition	–0.07	0.04	[−0.15, 0.01]	–0.15	–1.63	0.11	–0.05	0.04	[−0.13, 0.03]	–0.14	–1.32	0.19
Status/power	0.12	0.05	[0.03, 0.21]	0.27	2.61	0.01	0.08	0.06	[−0.04, 0.20]	0.13	1.33	0.19
Environmental exploration	0.02	0.03	[−0.04, 0.09]	0.07	0.73	0.47	0.02	0.03	[−0.04, 0.08]	0.06	0.61	0.54
Food selection	0.01	0.02	[−0.03, 0.06]	0.05	0.57	0.57	0.02	0.02	[−0.03, 0.06]	0.07	0.84	0.41
Food acquisition	0.01	0.04	[−0.06, 0.08]	0.03	0.36	0.72	0.09	0.04	[0.01, 0.16]	0.23	2.31	0.02
Parent-offspring conflict	–0.03	0.03	[−0.10, 0.03]	–0.10	–1.02	0.31	0.02	0.03	[−0.04, 0.08]	0.06	0.65	0.52
Kinship	0.01	0.04	[−0.07, 0.09]	0.01	0.16	0.87	0.09	0.05	[−0.01, 0.19]	0.16	1.73	0.09
Mate attraction	0.12	0.04	[0.04, 0.19]	0.30	3.14	0.00	0.15	0.05	[0.06, 0.25]	0.31	3.10	0.00
Mate retention	0.01	0.04	[−0.07, 0.08]	0.01	0.15	0.88	0.03	0.04	[−0.06, 0.12]	0.06	0.69	0.49

*Males R^2^ = 0.31 (p = 0.00). Females R^2^ = 0.21 (p = 0.00).*

## Discussion

The aim of the present study falls into two main objectives: (1) to analyze sex differences in risk perception, expected benefits and risk propensity in the evolutionary domains of risk, and (2) to test the predictive value of each of the risk contexts of the ERS in the evolutionary domains over the engagement in risk-taking behaviors in female and male adolescents. In relation to sex differences in the evolutionary domains in each of the risk contexts of the ERS, differences between male and female adolescents were found mainly in risk propensity (six of the ten evolutionary domains), followed by risk perception (four of the ten evolutionary domains) and, lastly, expected benefits (two of the ten evolutionary domains). More specifically, male adolescents expressed lower risk perception in environmental exploration and kinship, whereas female adolescents perceived less risk in mate retention and parent-offspring conflict. Moreover, male adolescents expected more benefits than female adolescents in environmental exploration and mate attraction, and showed higher propensity to take risks in between-group competition, within-group competition, status/power, environmental exploration, food acquisition and mate attraction. In general, results from the present study are similar to those found in previous research, which show that males tend to express lower risk perception and higher expected benefits, as well as higher propensity to take risks (e.g., Byrnes et al., 1999; Rhodes and Pivik, 2011; Grevenstein et al., 2015). Additionally, results are also in line with sex differences in the adaptive value of risk-taking behaviors, with risk-taking being mainly a matter for males (Trivers, 1972; Bird et al., 2001; Fessler et al., 2014; Archer, 2019).

Additionally, despite the sex differences found in some of the evolutionary domains of risk, no sex differences were found between male and female adolescents in the participation in risk-taking behaviors measured through the RBQ. Some authors feel reluctant to studies that analyze sex differences in risk-taking behaviors, mainly because such studies tend to use measurements that assess risk-taking behaviors that are mainly male-related (Morgenroth et al., 2018; Rolison and Shenton, 2019). In this sense, the RBQ assesses a wide variety of risk-taking behaviors under one single dimension, so the male bias found in risk-taking behaviors might be attenuated in such single dimension of risk-taking behaviors. However, it would be necessary to analyze to which extent risk-taking behaviors measured by the RBQ are bias-free.

With respect to the multiple regression models, all models of risk context of the ERS, except for the risk-perception model in female adolescents, predicted engagement in risk-taking behaviors, in line with previous research (e.g., MacPherson et al., 2010; [Bibr B6][Bibr B3]). More specifically, the context of expected benefits in the evolutionary domains was the model which explained the highest variance of risk-taking behaviors, both in female and male adolescents, as it has been found in previous research (Cauffman et al., 2010; Mantzouranis and Zimmermann, 2010). In general, these results suggest that risk-taking behaviors in adolescents are aimed in part at reaching evolutionary goals in the field of survival and reproduction. Results also demonstrate that female and male adolescents show both differences and similarities in the influence of the risk context of the ERS over risk-taking behaviors. Moreover, it is worth mentioning that risk perception in within-group competition and parent-offspring conflict predicted higher engagement in risk-taking behaviors. This finding suggests that considering the costs of taking risks not only reduces the probability of taking part in risk-taking behaviors, but it also may promote engagement in dangerous behaviors, as it has been previously seen (Hatfield and Fernandes, 2009). Results obtained from each of the evolutionary domains of risk are further discussed below.

When it comes to the domains related to intergroup and intragroup conflicts, male adolescents were more prone to risk than female adolescents in between-group competition, within-group competition, and status/power. Such higher propensity in male adolescents in these evolutionary domains could reflect a higher intrasexual competition compared to females (Anderson et al., 2015; Puts et al., 2016; Hess and Hagen, 2019). In fact, although the intensity of within-group competition tends to be similar between males and females (Hess and Hagen, 2019), results obtained show that male adolescents were more prone to take risks in this domain. Moreover, in male adolescents, risk perception in status/power predicted lower engagement in risk-taking behaviors, while risk propensity in status/power showed the opposite effect. These findings are in line with the core value of the domain of status/power in males (Wilson and Daly, 1985; Ellis et al., 2012; Archer, 2019; Buss and Schmitt, 2019). In fact, adolescents’ concern about avoiding social rejection and losing social status could protect them against participating in risky behaviors (Tomova et al., 2021). Thus, the negative effect of risk perception in status/power over risk-taking behaviors could protect against risk-taking behaviors in male adolescents. On the other hand, risk perception in within-group competition and risk-propensity in between-group competition predicted higher engagement in risk-taking behaviors in male adolescents. These results also demonstrate the functional value of risk-taking behaviors in inter-male competition (Ellis et al., 2012; Puts et al., 2016; Archer, 2019; Hess and Hagen, 2019).

Environmental exploration was the only evolutionary domain that showed sex differences in the three risk contexts of the ERS; males perceived less risk, expected more benefits, and expressed higher propensity to take risks compared to female adolescents. These sex differences in environmental exploration could be explained by the sex differences in labor during the Pleistocene, in which men were mainly engaged in hunting (which required more mobility and navigation), while women took care of their offspring and gathered plant food (which required less mobility and navigation) (Silverman and Choi, 2016).

In relation to the evolutionary domains associated with food, male adolescents showed higher risk propensity in food acquisition compared to female adolescents. The domain of food acquisition is characterized by a preference for food quantity over food quality, regardless of consuming spoiled food. Therefore, such lower propensity of female adolescents to ingest spoiled food would be in line with their higher expression of disgust sensitivity compared to males (Tybur et al., 2009). In fact, females tend to reject contact with food that could be contaminated more than males (García-Gómez et al., 2020), which could also be related to females’ higher risk of transmitting diseases to their offspring through food habits (Al-Shawaf et al., 2018). Furthermore, food acquisition also showed to be associated with risk-taking behaviors, in both male and female adolescents. In females, more propensity to take risks in food acquisition predicted higher engagement in risk-taking behaviors; while in males, expected benefits in food acquisition predicted higher engagement in risk-taking behaviors. Given that the domain of food acquisition is related to disgust, its inhibition can promote perceiving benefits and propensity in consuming food that is potentially harmful, especially in acute hunger or thirst situations (Oaten et al., 2009). Moreover, it has been observed that disgust is negatively related to sexual risk practices (e.g., promiscuity), substance use, and social transgression (e.g., lying, cheating or stealing) (Tybur et al., 2009; Oosterhoff and Shook, 2017). Therefore, we suggest that disgust inhibition could be the mechanism through which food acquisition promotes engagement in risk-taking behaviors.

Expected benefits in food selection predicted lower engagement in risk-taking behaviors in male adolescents. Thus, the perception of benefits in selecting high-quality food could act as a protective factor against risk-taking behaviors in male adolescents. In fact, a positive relationship between consuming low-quality food and engaging in risk-taking behaviors was previously found (Bruckauf and Walsh, 2018). This negative association between food selection and risk-taking behaviors could be due in part to the negative effects of the risk-taking behaviors measured with the RBQ (e.g., unsafe sexual practices, substance use or self-injurious behaviors) on individual health. By contrast, considering the benefits of taking risks in the domain of food selection would have positive effects on individual health.

In relation to parent-offspring conflict, female adolescents, compared to male adolescents, showed lower risk perception regarding the potential conflicts that could arise from their demands of parental investment. Additionally, higher risk perception in parent-offspring conflict predicted higher engagement in risk-taking behaviors in male adolescents. One possible explanation for these findings could be related with the world economic recession of 2008, which created a harsh and unpredictable environment for great part of the Spanish society. Specifically, the economic crisis of 2008 drastically increased unemployment rates in Spain, and such levels have not yet returned to the levels seen before the economic recession (Instituto Nacional de Estadística [INE], 2021). In fact, Durante et al. (2015) found that in adverse economic situations, parents tend to invest more in female offspring than male offspring because they perceive it is more likely for females to have children in such conditions. Therefore, the economic recession could have increased differences between male and female adolescents in parental investment, given the higher willingness of parents to invest resources in female offspring in harsh and unpredictable conditions. Finally, the economic recession could have also resulted in a higher risk for obtaining resources through parental investment in male adolescents. The final consequence is male adolescents’ need to take part in risk-taking behaviors to access such resources, something which has been demonstrated in bullying behaviors (Volk et al., 2012; Farrell and Dane, 2020).

With regard to the kinship domain, females perceived more risks in this domain than males, which could be explained in part by females’ higher tendency to avoid risks due to their role in offspring survival (Trivers, 1972; Fischer and Hills, 2012; Campbell et al., 2021). In fact, the kind of risky behaviors measured in the kinship domain along with the environmental exploration domain entail a higher level of danger compared to the risky behaviors measured in the other domains, which could explain such sex differences in both domains.

In the area of mating, male adolescents expected higher benefits and showed greater risk propensity in mate attraction, compared to female adolescents. This could be explained by the significant value of mate attraction for males when it comes to reproductive success, and due to being more oriented toward short-term relationships than females (Trivers, 1972; Wilson and Daly, 1985; Archer, 2019; Buss and Schmitt, 2019). Moreover, male adolescents’ expected benefits and risk propensity in mate attraction predicted higher engagement in risk-taking behaviors, which would be in line with the view of risk-taking as a cue for signaling abilities valued by females (Bird et al., 2001; Prokop and Pazda, 2020; Refaie and Mishra, 2020). Strikingly, female adolescents’ expected benefits and risk propensity in mate attraction also predicted higher engagement in risk-taking behaviors. This means that, in spite of its role in offspring survival and mate choice (Trivers, 1972; Archer, 2019), mate attraction also promotes risk-taking behaviors in female adolescents. In fact, this finding is in line with previous research which shows that females tend to engage in risk-taking behaviors in contexts of mate attraction (Arnocky and Vaillancourt, 2017).

On the other hand, in the domain of mate retention males showed higher risk perception than females. This finding is surprising if we look at males’ tendency toward short-term relationships compared to long-term relationships (Buss and Schmitt, 2019). However, it was recently found that males show higher tendency toward being unfaithful with their partner in long-term relationships (Apostolou, 2021). In addition, females are more prone to initiate divorce (Amato and Previti, 2003), being unfaithfulness one of the main reasons (Apostolou et al., 2019). Such higher disposition in males toward being unfaithful would increase the risk of breaking up the relationship, thus possibly explaining their higher risk perception in mate retention. Another possibility could be the greater variation in reproductive success in males along with the higher discrimination of females when choosing a mate (Trivers, 1972; Archer, 2019), which could lead males to give higher value to the risk of losing their current partner. A higher proportion of males than females could also explain males’ higher risk perception in mate retention (i.e., male-biased sex ratio) (Buss and Schmitt, 2019). Because pregnancy and nursing reduce females’ availability, males would value more their current partner due to their greater difficulty to find a new partner who can conceive. In sum, these observations could explain why males perceived the risks in mate retention domain to a greater extent in comparison to females.

### Alternative Explanations

The present study is based on an evolutionary approach to explain sex differences in risk perception, expected benefits and risk propensity in the evolutionary domains and the predictive effect of these domains over risk-taking behaviors in adolescents. However, findings from the present study could also be explained through other theoretical frameworks. One of these theoretical approaches, which is based on the study of risk-taking behaviors, is the Problem Behavior Theory (PBT) (Jessor, 2014). The PBT analyses the fundamental social-psychological processes involved in risk-taking behaviors, which act as protective and risk factors. This theory considers that risk-taking behaviors might play a key role in adolescents’ achievements of short-term goals. The PBT considers risk-taking behaviors as socially learned behaviors that help adolescents face frustration or failure, find similarities with peer groups, and as a way of opposing social norms. But above all, these behaviors indicate their transition to adulthood (Jessor, 2017). Nevertheless, these causes behind risk-taking behaviors in adolescents are not in conflict with the evolutionary model suggested in the present study, which is more oriented toward the ultimate causes of these behaviors (i.e., survival and reproduction). In short, the role of risk perception, expected benefits and risk propensity over risk-taking behaviors might be in part more related to other variables that are closer to the ones suggested by the PBT.

Moreover, in relation to the sex differences found in the present study, it is possible that other factors such as gender norms could explain these differences (Heise et al., 2019). Adolescence is considered a key stage in the “social production of gender,” where identity differences take form, as well as roles and norms between female and male adolescents (Kågesten et al., 2016; Weber et al., 2019). As a result, adolescents might be influenced to socialize in certain ways to take risks based on gender. Under such approach about gender norms, society would tend to promote certain masculinity constructs in male adolescents that are related to the search for status/power and mating, which would legitimate and reward them through risk-taking behaviors (Kennedy et al., 2020); the opposite would take place in the case of females (Heise et al., 2019). Furthermore, from this approach on gender norms, the concept of *sexual double standard* has appeared, which refers to the moral demands of higher sexual restriction in females to control their sexuality and reproduction, while sexual freedom is broadly allowed in males (Crawford and Popp, 2003). However, the present study has shown the predictive value of expected benefits and risk propensity in mate attraction over risk-taking behaviors in female adolescents, and which would contradict the hypothesis of *sexual double standard*. These findings are more in line with the evolutionary approach, which assumes that females would tend to take part in risky behaviors to reach their mating goals (Arnocky and Vaillancourt, 2017).

Finally, it must be mentioned that findings from the present study might be explained by other variables related to risk-taking behaviors, for example socioeconomic (Delker et al., 2018). In this sense, the relation found between the evolutionary domains of food selection and food acquisition with risk-taking behaviors could be mediated by socioeconomic status. In fact, a relation between parental socioeconomic status during childhood and unhealthy risk-taking behaviors such as smoking and drinking alcohol, as well as fast food intake was found (Marttila-Tornio et al., 2020). Moreover, this propensity to take more risks in people of low socioeconomic status could be a rational tactic of social competition in their environment. In fact, it has been demonstrated that risk-taking behaviors, including extreme behaviors like homicide, can be a form of social competition in environments of low economic inequality (Wilson and Daly, 1997). As a result, participants’ socioeconomic status could be a key variable in the results obtained in the present study.

### Limitations

In spite of the predictive terminology used in the present study, results show no causal effects. Moreover, it should be mentioned that answers to the RBQ and ERS could be affected by the level of honesty of participants, due to some items containing sensitive content (Tourangeau and Yan, 2007). Thus, it is possible that the sensitive content of some items along with the classroom setting where the study was carried out enhanced socially desirable responding. As a result, and although the confidentiality of answers was guaranteed to participants, low scores obtained in the RBQ may not indicate the real level of risk-taking behaviors in adolescents. Furthermore, present study was carried out with adolescent students during classroom time. Thus, due to time constraints we could not expose each participant to the three risk contexts of the ERS. As a consequence, intraindividual differences between the risk contexts of the ERS could not be analyzed. Nevertheless, although results might have been different, the sample size for each risk context provides sufficient validity to analyze differences in risk perception, expected benefits and risk propensity in adolescents.

### Further Research

As it has been mentioned, results obtained in the present study are correlational. It is necessary, therefore, to carry out experimental research in order to provide a causal relationship between the evolutionary specific domains and risk-taking behaviors in adolescents. Likewise, and based on the comments in the “Limitations” section, it would be appropriate to carry out a future line of research with an intrasubject design. This way, each participant would go through the three contexts of the ERS, thus allowing to find the intraindividual differences in risk perception, expected benefits and propensity in the evolutionary domains of risk. Moreover, mate attraction was also found to predict higher engagement in risk-taking behaviors in female adolescents, so, it would be useful to analyze male adolescents’ attitudes toward this female-mate attraction risky strategies, which could also make mate choice a male issue. Finally, regarding the effects found of food domains (i.e., food acquisition and food selection) on risk-taking behaviors, further investigation would be needed to determinate how healthy diet interventions could reduce engagement in risk-taking behaviors, and particularly, intervention programs whose main objective would improving healthy diets in adolescents, as they could also be effective in reducing adolescent engagement in risk-taking behaviors.

### Implications

Notwithstanding its limitations, the present study has both theoretical and practical implications. At a theoretical level, findings demonstrate in part the adaptive value of risk-taking behaviors in adolescents. This means that, despite their negative consequences, risky behaviors can have potential benefits for both female and male adolescents. In fact, the expected benefits model was the one that explained the highest variance in risk-taking behaviors, both in males and females. These findings contradict traditional models that explain risk-taking behaviors through psychopathological perspectives. From an evolutionary view, adolescents who take risks do not necessarily suffer mental deficiencies derived from stressful environments. Conversely, we propose considering risk-taking behaviors as adaptive strategies through which adolescents can reach their survival and reproductive goals (Ellis et al., 2012; Machluf and Bjorklund, 2015).

At a practical level, intervention programs focused on risk-taking behaviors should consider the functional value of risky behaviors in adolescents. Generally, these programs aim at reducing behaviors that, although harmful, can have potential benefits in adolescents. Therefore, such programs should promote alternative behaviors for adolescents through which they can reach their vital goals. Intervention programs should also be cautious on putting too much emphasis on the potential costs of these type of behaviors, given the finding that risk perception could promote engagement in risk-taking behaviors, especially in males. Along with proposing behavioral alternatives to risk assumption, intervention programs should tackle risk management in those behaviors that cannot be replaced with other less dangerous behaviors, and which imply potential benefits in adolescents. For example, given the functional value of sexual practices among adolescents, programs should aim to help adolescents manage and understand the relative costs related to such behaviors (i.e., promoting the use of contraceptive measures), instead of carrying out an intervention based on zero tolerance. Finally, considering an evolutionary view implies the need of designing more specific programs that would consider both the evolutionary specific domains and sex. Given sex differences in risk-taking, intervention programs need to be designed specifically for males and females. For example, programs would beneficiate from considering the protective value of risk perception in status/power in male adolescents, through the designing of interventions that would trigger awareness on the potential risks in this domain if male adolescents took part in risk-taking behaviors.

## Conclusion

Sex difference were found in perception or risk, expected benefits and the propensity to take risks in the evolutionary domains, which could reflect differences between female and male adolescents in the adaptive value of these domains. Moreover, findings in the regression models suggest that risk-taking behaviors can be conceptualized as adaptive behaviors with potential costs but also with potential benefits for adolescents. As a result, and contrary to a psychopathological perspective, risk-taking behaviors can act as rational strategies in adolescents. Therefore, we consider that intervention programs should be designed by applying an evolutionary standpoint with the purpose of reducing risk-taking behaviors that are dangerous to the adolescents’ health; mainly by considering both sex differences and alternative behaviors through which adolescents can reach their survival and reproduction goals.

## Data Availability Statement

The raw data supporting the conclusions of this article will be made available by the authors, without undue reservation.

## Ethics Statement

The studies involving human participants were reviewed and approved by Ethical Committee on Experimentation from the University of Málaga (CEUMA) (Registry number: 45-2018-H). Written informed consent to participate in this study was provided by the participants’ legal guardian/next of kin.

## Author Contributions

JS-R participated in the collection and analysis of data, and drafted the manuscript. LG-J and IH-M contributed to the conception and design of the work. NP-B collected the data and participated in the drafting of the manuscript. LG-J reviewed drafts of the manuscripts and approved the final manuscript. All authors contributed to the article and approved the submitted version.

## Conflict of Interest

The authors declare that the research was conducted in the absence of any commercial or financial relationships that could be construed as a potential conflict of interest.

## Publisher’s Note

All claims expressed in this article are solely those of the authors and do not necessarily represent those of their affiliated organizations, or those of the publisher, the editors and the reviewers. Any product that may be evaluated in this article, or claim that may be made by its manufacturer, is not guaranteed or endorsed by the publisher.

## References

[B1] Aklin W. M. Lejuez C. W. Zvolensky M. J. Kahler C. W. Gwadz M. (2005). Evaluation of behavioral measures of risk-taking propensity with inner city adolescents. *Behav. Res. Ther.* 43 215–228. 10.1016/j.brat.2003.12.007 15629751

[B2] Al-Shawaf L. Lewis D. M. G. Buss D. M. (2018). Sex differences in disgust: why are women more easily disgusted than men? *Emot. Rev.* 10 149–160.

[B3] Altikriti S. Nedelec J. L. Silver I. (2021). The role of arrest risk perception formation in the association between psychopathy and aggressive offending. *Youth Violence Juv. Justice*. 19 402–422. 10.1177/15412040211029991

[B4] Amato P. R. Previti D. (2003). People’s reasons for divorcing: gender, social class, the life course, and adjustment. *J. Fam. Issues* 24 602–626. 10.1177/0192513X03024005002

[B5] Anderson C. Angus J. Hildreth D. Howland L. (2015). Is the desire for status a fundamental human motive? A review of the empirical literature. *Psychol. Bull.* 141 574–601. 10.1037/a0038781 25774679

[B6] Andrews J. L. Mills K. L. Flournoy J. C. Flannery J. E. Mobasser A. Garrett R. (2020). Expectations of social consequences impact anticipated involvement in health-risk behavior during adolescence. *J. Res. Adolesc*. 30 1008–1024. 10.1111/jora.12576 32910510 PMC8494461

[B7] Apicella C. L. Crittenden A. N. Tobolsky V. A. (2017). Hunter-gatherer males are more risk-seeking than females, even in late childhood. *Evol. Hum. Behav*. 38 592–603. 10.1016/j.evolhumbehav.2017.01.003

[B8] Apostolou M. (2021). Plurality in mating: exploring the occurrence and contingencies of mating strategies. *Pers. Individ. Dif.* 175:110689. 10.1016/j.paid.2021.110689

[B9] Apostolou M. Constantinou C. Anagnostopoulos S. (2019). Reasons that could lead people to divorce in an evolutionary perspective: evidence from Cyprus. *J. Divorce Remarriage* 60 27–46. 10.1080/10502556.2018.1469333

[B10] Archer J. (2004). Sex differences in aggression in real-world settings: a meta-analytic review. *Rev. Gen. Psychol*. 8 291–322. 10.1037/1089-2680.8.4.291

[B11] Archer J. (2019). The reality and evolutionary significance of human psychological sex differences. *Biol. Rev*. 94 1381–1415. 10.1111/brv.12507 30892813

[B12] Arnocky S. Vaillancourt T. (2017). “Sexual competition among women: a review of the theory and supporting evidence,” in *The Oxford Handbook of Women and Competition*, ed. Fisher M. L. (New York, NY: Oxford University Press), 25–39. 10.1016/j.yfrne.2013.07.001

[B13] Auerbach R. P. Gardiner C. K. (2012). Moving beyond the trait conceptualization of self-esteem: the prospective effect of impulsiveness, coping, and risky behavior engagement. *Behav. Res. Ther.* 50 596–603. 10.1016/j.brat.2012.06.002 22835840

[B14] Bird R. B. Smith E. A. Bird D. W. (2001). The hunting handicap: costly signaling in human foraging strategies. *Behav. Ecol. Sociobiol*. 50 9–19. 10.1007/s002650100338

[B15] Blais A. Weber E. U. (2006). A domain-specific risk-taking (DOSPERT) scale for adult populations. *Judgm. Decis. Mak.* 1 33–47. 10.2466/09.03.PR0.114k15w2 24765720

[B16] Bruckauf Z. Walsh S. D. (2018). Adolescents’ multiple and individual risk behaviors: examining the link with excessive sugar consumption across 26 industrialized countries. *Soc. Sci. Med.* 216 133–141. 10.1016/j.socscimed.2018.08.029 30269866

[B17] Buss D. M. Schmitt D. P. (2019). Mate preferences and their behavioral manifestations. *Annu. Rev. Psychol.* 70 77–110. 10.1146/annurev-psych-010418-103408 30230999

[B18] Byrnes J. P. Miller D. C. Schafer W. D. (1999). Gender differences in risk taking: a meta-analysis. *Psychol. Bull.* 125 367–383. 10.1037/0033-2909.125.3.367

[B19] Campbell A. Copping L. T. Cross C. P. (2021). “Fear, sex differences and the ‘Staying alive’ hypothesis,” in *Sex Differences in Fear Response. An Evolutionary Perspective*, (Cham: Springer), 1–6. 10.1007/978-3-030-65280-7_1

[B20] Campbell R. Wright C. Hickman M. Kipping R. R. Smith M. Pouliou T. (2020). Multiple risk behaviour in adolescence is associated with substantial adverse health and social outcomes in early adulthood: findings from a prospective birth cohort study. *Prev. Med*. 138:106157. 10.1016/j.ypmed.2020.106157 32473267 PMC7378566

[B21] Carlson G. C. Duckworth M. P. (2019). Sexual victimization and benefit expectations of risky behavior among female college students. *J. Interpers. Violence* 34 1543–1562. 10.1177/0886260516651626 27271982

[B22] Cauffman E. Shulman E. P. Steinberg L. Claus E. Banich M. T. Graham S. (2010). Age differences in affective decision making as indexed by performance on the Iowa Gambling Task. *Dev. Psychol*. 46 193–207. 10.1037/a0016128 20053017

[B23] Crawford M. Popp D. (2003). Sexual double standards: a review and methodological critique of two decades of research. *J. Sex Res.* 40 13–26. 10.1080/00224490309552163 12806528

[B24] Delker B. C. Bernstein R. E. Laurent H. K. (2018). Out of harm’s way: secure versus insecure–disorganized attachment predicts less adolescent risk taking related to childhood poverty. *Dev. Psychopathol*. 30 283–296. 10.1017/S0954579417000621 28508736

[B25] Dhami M. K. Mandel D. R. (2012). Crime as risk taking. *Psychol. Crime Law* 18 389–403. 10.1080/1068316X.2010.498423

[B26] Durante K. M. Griskevicius V. Redden J. P. White A. E. (2015). Spending on daughters versus sons in economic recessions. *J. Consum. Res.* 42 435–457. 10.1093/jcr/ucv023

[B27] Ellis B. J. (2012). “Risky adolescent behavior: an evolutionary perspective,” in *Adolescent Identity: Evolutionary, Cultural and Developmental Perspectives*, ed. Hewlett B. L. (New York, NY: Routledge), 40–72. 10.4324/9780203102381

[B28] Ellis B. J. Del Giudice M. Dishion T. J. Figueredo A. J. Gray P. Griskevicius V. (2012). The evolutionary basis of risky adolescent behavior: implications for science, policy, and practice. *Dev. Psychol.* 48 598–623. 10.1037/a0026220 22122473

[B29] Essau C. A. (2004). Risk-taking behaviour among German adolescents. *J. Youth Stud.* 7 499–512. 10.1080/1367626042000315248

[B30] Farrell A. H. Dane A. V. (2020). Bullying, victimization, and prosocial resource control strategies: differential relations with dominance and alliance formation. *Evol. Behav. Sci.* 14 270–283. 10.1037/ebs0000178

[B31] Fessler D. M. Tiokhin L. B. Holbrook C. Gervais M. M. Snyder J. K. (2014). Foundations of the crazy bastard hypothesis: nonviolent physical risk-taking enhances conceptualized formidability. *Evol. Hum. Behav.* 35 26–33. 10.1016/j.evolhumbehav.2013.09.003

[B32] Figner B. Weber E. U. (2011). Who takes risks when and why? Determinants of risk taking. *Curr. Dir. Psychol. Sci.* 20 211–216. 10.1177/0963721411415790

[B33] Fischer D. Hills T. T. (2012). The baby effect and young male syndrome: social influences on cooperative risk-taking in women and men. *Evol. Hum. Behav.* 33 530–536. 10.1016/j.evolhumbehav.2012.01.006

[B34] García-Gómez L. Romero-Rebollar C. Hartmann C. Siegrist M. Ferreira G. Gutierrez-Aguilar R. (2020). Food disgust scale: Spanish version. *Front. Pychol.* 11:165. 10.3389/fpsyg.2020.00165 32116959 PMC7020908

[B35] Gibbons F. X. Houlihan A. E. Gerrard M. (2009). Reason and reaction: the utility of a dual-focus, dual-processing perspective on promotion and prevention of adolescent health risk behaviour. *Br. J. Health Psychol.* 14 231–248. 10.1348/135910708X376640 19026095

[B36] Goldberg J. H. Halpern-Felsher B. L. Millstein S. G. (2002). Beyond invulnerability: the importance of benefits in adolescents’ decision to drink alcohol. *Heal. Psychol.* 21 477–484. 10.1037/0278-6133.21.5.477 12211515

[B37] Grevenstein D. Nagy E. Kroeninger-Jungaberle H. (2015). Development of risk perception and substance use of tobacco, alcohol and cannabis among adolescents and emerging adults: evidence of directional influences. *Subst. Use Misuse* 50 376–386. 10.3109/10826084.2014.984847 25496046

[B38] Hames R. (2016). “Kin selection,” in *The Handbook of Evolutionary Psychology: Foundations*, ed. Buss D. M. (Hoboken, NJ: Wiley), 505–523.

[B39] Hamilton W. D. (1964). The genetical evolution of social behaviour. *J. Theor. Biol.* 7 1–16. 10.1016/0022-5193(64)90038-4 5875341

[B40] Hammond C. J. Krishnan-Sarin S. Mayes L. C. Potenza M. N. Crowley M. J. (2020). Associations of cannabis- and tobacco-related problem severity with reward and punishment sensitivity and impulsivity in adolescent daily cigarette smokers. *Int. J. Ment. Health Addiction* 19, 1963–1979. 10.1007/s11469-020-00292-2 PMC8735161 35002579

[B41] Hanoch Y. Johnson J. G. Wilke A. (2006). Domain specificity in experimental measures and participant recruitment: an application to risk-taking behavior. *Psychol. Sci.* 17 300–304. 10.1111/j.1467-9280.2006.01702.x 16623686

[B42] Hatfield J. Fernandes R. (2009). The role of risk-propensity in the risky driving of younger drivers. *Accid. Anal. Prev.* 41 25–35. 10.1016/j.aap.2008.08.023 19114134

[B43] Heise L. Greene M. E. Opper N. Stavropoulou M. Harper C. Nascimento M. (2019). Gender inequality and restrictive gender norms: framing the challenges to health. *Lancet* 393 2440–2454. 10.1016/S0140-6736(19)30652-X 31155275

[B44] Henrich J. Muthukrishna M. (2021). The origins and psychology of human cooperation. *Annu. Rev. Psychol.* 72 207–240. 10.1146/annurev-psych-081920-042106 33006924

[B45] Hess N. H. Hagen E. H. (2019). “Gossip, reputation, and friendship in within-group competition,” in *The Oxford Handbook of Gossip and Reputation*, eds Giardini F. Wittek R. (New York, NY: Oxford University Press), 275–302. 10.1093/oxfordhb/9780190494087.013.15

[B46] Instituto Nacional de Estadística [INE] (2021). *Encuesta de Población Activa (EPA).* Available online at: https://www.ine.es/prensa/epa_tabla.htm (Accessed August 15, 2021)

[B47] Jessor R. (2014). “Problem behavior theory: a half-century of research on adolescent behavior and development,” in *The Developmental Science of Adolescence: History Through Autobiography*, eds Lerner R. M. Petersen A. C. Silbereisen R. K. Brooks-Gunn J. (New York, NY: Psychology Press), 239–256. 10.1007/s10964-018-0811-z

[B48] Jessor R. (2017). *Problem Behavior Theory and Adolescent Health: The Collected Works of Richard Jessor.* Cham: Springer International Publishing, 10.1007/978-3-319-51349-2

[B49] Kågesten A. Gibbs S. Blum R. W. Moreau C. Chandra-Mouli V. Herbert A. (2016). Understanding factors that shape gender attitudes in early adolescence globally: a mixed-methods systematic review. *PLoS One* 11:e0157805. 10.1371/journal.pone.0157805 27341206 PMC4920358

[B50] Kennedy E. Binder G. Humphries-Waa K. Tidhar T. Cini K. Comrie-Thomson L. (2020). Gender inequalities in health and wellbeing across the first two decades of life: an analysis of 40 low-income and middle-income countries in the Asia-Pacific region. *Lancet Global Health* 8 e1473–e1488. 10.1016/S2214-109X(20)30354-5 33091371

[B51] Kershaw T. S. Ethier K. A. Niccolai L. M. Lewis J. B. Ickovics J. R. (2003). Misperceived risk among female adolescents: social and psychological factors associated with sexual risk accuracy. *Heal. Psychol.* 22 523–532. 10.1037/0278-6133.22.5.523 14570536

[B52] Lejuez C. W. Aklin W. M. Zvolensky M. J. Pedulla C. M. (2003). Evaluation of the Balloon Analogue Risk Task (BART) as a predictor of adolescent real-world risk-taking behaviours. *J. Adolesc.* 26 475–479. 10.1016/S0140-1971(03)00036-8 12887935

[B53] Lejuez C. W. Aklin W. Daughters S. Zvolensky M. Kahler C. Gwadz M. (2007). Reliability and validity of the youth version of the balloon analogue risk task (BART–Y) in the assessment of risk-taking behavior among inner-city adolescents. *J. Clin. Child Adolesc. Psychol.* 36 106–111. 10.1080/15374410709336573 17206886

[B54] Luoto S. Varella M. A. C. (2021). Pandemic leadership: sex differences and their evolutionary–developmental origins. *Front. Psychol.* 12:618. 10.3389/fpsyg.2021.633862 33815218 PMC8015803

[B55] Machluf K. Bjorklund D. F. (2015). “Understanding risk-taking behavior: insights from evolutionary psychology,” in *Emerging Trends in the Social and Behavioral Sciences: An Interdisciplinary, Searchable, and Linkable Resource*, eds Kosslyn S. M. Scott R. A. (Hoboken, NJ: Wiley), 1–15.

[B56] MacPherson L. Magidson J. F. Reynolds E. K. Kahler C. W. Lejuez C. W. (2010). Changes in sensation seeking and risk-taking propensity predict increases in alcohol use among early adolescents. *Alcohol. Clin. Exp. Res.* 34 1400–1408. 10.1111/j.1530-0277.2010.01223.x 20491737 PMC3123723

[B57] Mantzouranis G. Zimmermann G. (2010). Taking risks, worth it? Risk behaviours and perceiving their risks among adolescents. *Neuropsychiatr. Enfance. Adolesc.* 58 488–494. 10.1016/j.neurenf.2010.02.003

[B58] Martínez-Montilla J. M. Mercken L. Lima-Serrano M. de Vries H. Lima-Rodríguez J. S. (2020). Why are Spanish adolescents binge drinkers? Focus group with adolescents and parents. *Int. J. Environ. Res. Public Health* 17:3551. 10.3390/ijerph17103551 32438735 PMC7277407

[B59] Marttila-Tornio K. Männikkö N. Ruotsalainen H. Miettunen J. Kääriäinen M. (2020). Lower parental socioeconomic status in childhood and adolescence predicts unhealthy health behaviour patterns in adolescence in Northern Finland. *Scand. J. Caring Sc*i. 35, 742–752. 10.1111/scs.12888 32666577

[B60] Maslowsky J. Keating D. P. Monk C. S. Schulenberg J. (2011). Planned versus unplanned risks: neurocognitive predictors of subtypes of adolescents’ risk behavior. *Int. J. Behav. Dev.* 35 152–160. 10.1177/0165025410378069 22679340 PMC3367561

[B61] Morgenroth T. Fine C. Ryan M. K. Genat A. E. (2018). Sex, drugs, and reckless driving: are measures biased toward identifying risk-taking in men? *Soc. Psychol. Pers.* 9 744–753. 10.1177/1948550617722833

[B62] Oaten M. Stevenson R. J. Case T. I. (2009). Disgust as a disease-avoidance mechanism. *Psychol. Bull.* 135 303–321. 10.1037/a0014823 19254082

[B63] Oosterhoff B. Shook N. J. (2017). From drug laws to recreational substance use: the adaptationist role of disgust sensitivity. *Pers. Individ. Dif.* 104 544–553. 10.1016/j.paid.2016.09.015

[B64] Parsons J. T. Halkitis P. N. Bimbi D. Borkowski T. (2000). Perceptions of the benefits and costs associated with condom use and unprotected sex among late adolescent college students. *J. Adolesc.* 23 377–391. 10.1006/jado.2000.0326 10936012

[B65] Prokop P. Pazda A. (2020). Individual differences in preference for risky behaviors during courtship. *Scand. J. Psychol.* 61 560–564. 10.1111/sjop.12628 32103513

[B66] Puts D. A. Bailey D. H. Reno P. L. (2016). “Contest competition in men,” in *The Handbook of Evolutionary Psychology: Foundations*, ed. Buss D. M. (Hoboken, NJ: Wiley), 385–402.

[B67] Refaie N. Mishra S. (2020). Embodied capital and risk-related traits, attitudes, behaviors, and outcomes: an exploratory examination of attractiveness, cognitive ability, and physical ability. *Soc. Psychol. Personal. Sci.* 11 949–964. 10.1177/1948550619882036

[B68] Reniers R. L. Beavan A. Keogan L. Furneaux A. Mayhew S. Wood S. J. (2017). Is it all in the reward? Peers influence risk-taking behaviour in young adulthood. *Br. J. Psychol.* 108 276–295. 10.1111/bjop.12195 26990740

[B69] Reniers R. L. Murphy L. Lin A. Bartolomé S. P. Wood S. J. (2016). Risk perception and risk-taking behaviour during adolescence: the influence of personality and gender. *PloS One* 11:e0153842. 10.1371/journal.pone.0153842 27100081 PMC4839773

[B70] Rhodes N. Pivik K. (2011). Age and gender differences in risky driving: the roles of positive affect and risk perception. *Accid. Anal. Prev.* 43 923–931. 10.1016/j.aap.2010.11.015 21376884

[B71] Rolison J. J. Shenton J. (2019). How much risk can you stomach? Individual differences in the tolerance of perceived risk across gender and risk domain. *J. Behav. Decis. Mak.* 33 63–85. 10.1002/bdm.2144

[B72] Rozin P. Todd P. M. (2016). “The evolutionary psychology of food intake and choice,” in *The Handbook of Evolutionary Psychology: Foundations”*, ed. Buss D. M. (Hoboken, NJ: Wiley), 183–205.

[B73] Rundmo T. Iversen H. (2004). Risk perception and driving behaviour among adolescents in two Norwegian counties before and after a traffic safety campaign. *Saf. Sci.* 42 1–21. 10.1016/S0925-7535(02)00047-4

[B74] Salas-Rodríguez J. Gómez-Jacinto L. Hombrados-Mendieta M. I. (2021). Life history theory: evolutionary mechanisms and gender role on risk-taking behaviors in young adults. *Pers. Individ. Dif.* 175:110752. 10.1016/j.paid.2021.110752

[B75] Schaller M. (2018). The parental care motivational system and why it matters (for everyone). *Curr. Dir. Psychol. Sci.* 27 295–301. 10.1177/0963721418767873

[B76] Silverman I. Choi J. (2016). “Spatial navigation and landscape preferences,” in *The Handbook of Evolutionary Psychology: Foundations*, ed. Buss D. M. (Hoboken, NJ: Wiley), 225–245.

[B77] Steinberg L. (2008). A social neuroscience perspective on adolescent risk-taking. *Dev. Rev.* 28 78–106. 10.1016/j.dr.2007.08.002 18509515 PMC2396566

[B78] Tomova L. Andrews J. L. Blakemore S. J. (2021). The importanece of belonging and the avoidance of social risk taking in adolescence. *Dev. Rev.* 61:100981. 10.1016/j.dr.2021.100981

[B79] Tooby J. Cosmides L. (2005). “Conceptual foundations of evolutionary psychology,” in *The Handbook of Evolutionary Psychology*, ed. Buss D. M. (Hoboken, NJ: Wiley), 5–67. 10.1002/9780470939376.ch1

[B80] Tourangeau R. Yan T. (2007). Sensitive questions in surveys. *Psychol. Bull.* 133 859–883. 10.1037/0033-2909.133.5.859 17723033

[B81] Trivers R. L. (1972). “Parental investment and sexual selection,” in *Sexual Selection and the Descent of Man*, ed. Campbell B. (Chicago, IL: Aldinc), 136–179. 10.4324/9781315129266-7

[B82] Tybur J. M. Lieberman D. Griskevicius V. (2009). Microbes, mating, and morality: individual differences in three functional domains of disgust. *J. Pers. Soc. Psychol.* 97 103–122. 10.1037/a0015474 19586243

[B83] Volk A. A. Camilleri J. A. Dane A. V. Marini Z. A. (2012). Is adolescent bullying an evolutionary adaptation? *Aggress. Behav.* 38 222–238. 10.1002/ab.21418 22331629

[B84] Warne R. T. (2014). A primer on multivariate analysis of variance (MANOVA) for behavioral scientists. *Pract. Assess. Res. Eval*. 19 1–10. 10.7275/sm63-7h70

[B85] Weber A. M. Cislaghi B. Meausoone V. Abdalla S. Mejía-Guevara I. Loftus P. (2019). Gender norms and health: insights from global survey data. *Lancet*. 393:10189. 10.1016/S0140-6736(19)30765-2 31155273

[B86] Weber E. U. Blais A. R. Betz N. E. (2002). A domain-specific risk-attitude scale: measuring risk perceptions and risk behaviors. *J. Behav. Decis. Mak.* 15 263–290. 10.1002/bdm.414

[B87] Widdice L. E. Cornell J. L. Liang W. Halpern-Felsher B. L. (2006). Having sex and condom use: potential risks and benefits reported by young, sexually inexperienced adolescents. *J. Adolesc. Heal.* 39 588–595. 10.1016/j.jadohealth.2006.03.016 16982395

[B88] Wilke A. Sherman A. Curdt B. Mondal S. Fitzgerald C. Kruger D. J. (2014). An evolutionary domain-specific risk scale. *Evol. Behav. Sci.* 8 123–141. 10.1037/ebs0000011

[B89] Willoughby T. Heffer T. Good M. Magnacca C. (2021). Is adolescence a time of heightened risk taking? An overview of types of risk-taking behaviors across age groups. *Dev. Rev.* 61:100980. 10.1016/j.dr.2021.100980

[B90] Wilson M. Daly M. (1985). Competitiveness, risk taking, and violence: the young male syndrome. *Ethol. Sociobiol.* 6 59–73. 10.1016/0162-3095(85)90041-X

[B91] Wilson M. Daly M. (1997). Life expectancy, economic inequality, homicide, and reproductive timing in Chicago neighbourhoods. *BMJ* 314 1271–1274. 9154035 10.1136/bmj.314.7089.1271 PMC2126620

